# Swallowing outcomes after tracheal resection and anastomosis: full versus mini infrahyoid laryngeal drop

**DOI:** 10.1007/s00405-024-08904-6

**Published:** 2024-08-29

**Authors:** Ahmed Abdel-Fattah ElSobki, Noha Ahmed El-Kholy, Eslam Hamed Elsayed Abdou, Reham A.E. Ibrahim, Ayman Amer, Mohamed El-Deeb, Mahmoud Elsaid Ibrahim Alsobky, Ahmed Negm

**Affiliations:** 1 Department of Otorhinolaryngology-Head & Neck Surgery, Faculty of Medicine, Mansoura University, El-Gomhoria Street, Mansoura, Dakahlia Governorate Egypt; 2 Department of Phoniatrics, Faculty of Medicine, Assiut University, Assiut, Egypt; 3 Department of Phoniatrics, Faculty of Medicine, Mansoura University, Mansoura, Egypt; 4 Department of Otorhinolaryngology-Head & Neck Surgery, Faculty of Medicine, Kafr El-Sheikh University, Kafr El-Sheikh, Egypt; 5 Department of Otorhinolaryngology-Head & Neck Surgery, Faculty of Medicine, Alazhar University, Damietta, Egypt; 6 Department of Otorhinolaryngology-Head & Neck Surgery, Faculty of Medicine, Misr University for Science and Technology, Mansoura, Egypt

**Keywords:** Suprahyoid, Infrahyoid, Laryngeal drop, Tracheal resection and anastomosis, Swallowing, Dysphagia

## Abstract

**Introduction:**

Tracheal resection anastomosis has been established as the definitive surgery for high grade postintubation subglottic stenosis. To achieve a relaxed tension-free anastomosis, various laryngeal release techniques were discussed in literature with potential effect on postoperative swallowing dysfunction. This study aims to compare the difference in swallowing outcomes following two methods of infrahyoid laryngeal release: with and without fracture of the superior thyroid horns.

**Methods:**

A retrospective cohort study was carried out at our tertiary referral hospitals including cases with grade III and IV subglottic stenosis treated by partial crico-tracheal resection with thyro-tracheal anastomosis. The patients were divided into two groups according to the method used in laryngeal release; mini infrahyoid release (group A) or infrahyoid full release (group B) where full means with fracture of the superior thyroid horn bilaterally while mini means their preservation. Swallowing assessment preoperatively and postoperatively was done by comparing swallowing dysfunction symptoms, Gugging swallowing screen (GUSS) score and fiberoptic endoscopic evaluation of swallowing (FEES) according to penetration aspiration scale (PAS).

**Results:**

A total of 71 patients were included; 46 in Group A and 25 in Group B. Clinical swallowing evaluation one week postoperatively showed statistically significant difference between the two groups being affected in 80.04% and 100% of patients in group A and B, respectively. The mean postoperative GUSS were 18 ± 1.32 in group A patients in comparison to 8.84 ± 5.18 in group B (*p*-value < 0.001). With FEES assessment, group A had full improvement of their swallowing abilities one month after the surgery while patients in group B had significantly lower PAS scores. Unfavourable scores for both the GUSS test and PAS were associated with increasing patients’ age in group B.

**Conclusion:**

In this retrospective cohort study, cases with mini infrahyoid laryngeal release had significantly better swallowing outcomes and full resolution of dysphagia in comparison to full laryngeal release. Also, full laryngeal release is associated with delayed resolution of swallowing difficulty in older patients. This point should be considered during preoperative patient selection and counselling.

## Introduction

Laryngotracheal stenosis involves narrowing of the upper airway and can be idiopathic, or due to traumatic, neoplastic or autoimmune causes. The most commonly reported cause is airway trauma caused by prolonged intubation with an average rate of postintubation stenosis ranging from10–22% [1–4]. Treatment of these challenging cases ranges from endoscopic lysis of the stenosis to major airway resection surgeries as tracheal or crico-tracheal resection [5, 6].

Tracheal resection anastomosis has been developed throughout years to safely perform a well-vascularized, low tension anastomosis with excellent outcomes especially in the cases of difficult lengthy stenosis [7]. To achieve this, various laryngotracheal release techniques had been discussed to reduce the anastomotic tension and facilitate closure of the anastomosis. These techniques include; sectioning of the suprahyoid and infrahyoid muscles and mediastinal release [8].

Although the release techniques allow for an easier approximation of the anastomotic ends, they involve disruption of the neighbouring pharyngeal muscles, suprahyoid and infrahyoid muscles as well as cartilaginous framework of the larynx and trachea. This is associated with supraglottic elongation and limitation of laryngeal elevation with adverse effects on swallowing and reported postoperative dysphagia and aspiration [9, 10].

Recently, studies have begun to investigate the impact of different laryngeal drop techniques on postoperative swallowing function [11]. This is critically important not only to predict patient and surgical factors associated with postoperative dysphagia, but also to guide appropriate laryngeal release technique and anticipate swallowing recovery. The current study aims to report the difference in swallowing outcomes following two methods of infrahyoid laryngeal drop: with and without fracture of the superior thyroid horns to detect the method with less postoperative dysphagia and faster recovery.

## Patients and methods

### Study design and patient characteristics

This retrospective cohort study was conducted at our tertiary referral hospitals over cases diagnosed with post intubation subglottic stenosis, Grade III and IV stenosis [12], and underwent partial crico-tracheal resection (PCTR) with thyro-tracheal anastomosis or lower tracheo-tracheal resection without cricoid involvement as a treatment of their condition. Only cases with single-stage surgery, without tracheostomy, were included in the study. Medical records of the cases over an eight-year period, from January 2016 till January 2024 were precisely revised.

Cases with subglottic stenosis due to other causes, other than prolonged intubation, as granulomas or malignancy and cases with incomplete records were excluded. Also, cases with preoperative swallowing problems and neurological affection were not included in the current study. If additional surgical technique was added to the crico-tracheal resection, as posterior cricoid split or cartilage grafting, the case was excluded from the study. Also, cases with impaired vocal cord mobility were not included. Approval of the ethical review board and written informed consents were obtained.

**Fig. 1:**
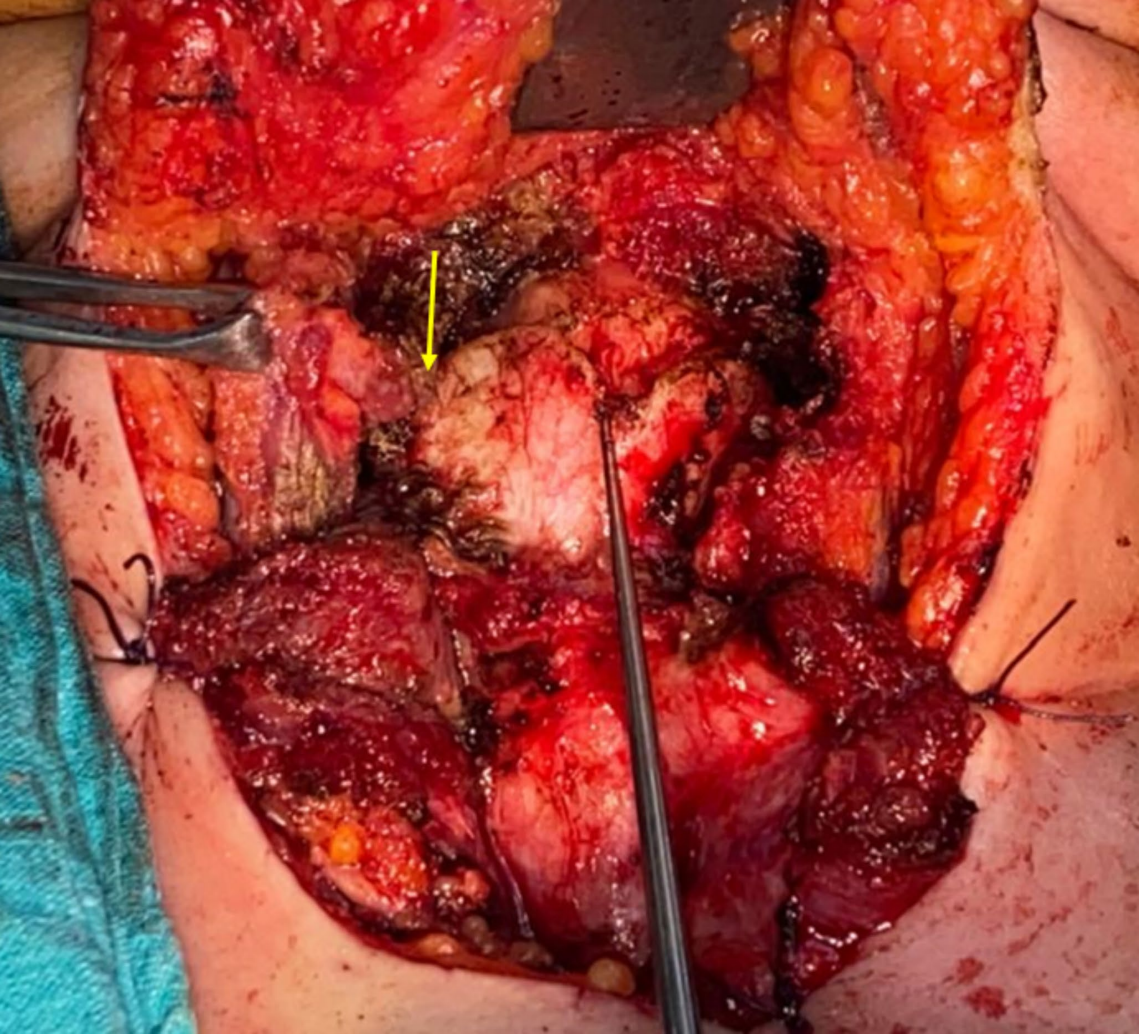
Demonstrates the step of infrahyoid laryngeal drop during a partial crico-tracheal resection surgery. Yellow arrow points to the line of fracture of the right superior thyroid horn with an acceptable release upon pulling down by a hook

All cases had mediastinal release with cranial mobilization together with infrahyoid laryngeal drop to achieve tension free closure and decrease the possibility of dehiscence. Laryngeal drop classifies the patients into two groups according to the degree of this drop, either mini (group A) or full (group B). Full drop means release of infrahyoid muscles together with fracture of the superior thyroid horn bilaterally while mini entails preservation of thyroid horns without fracture [13]. The choice between both was done intraoperatively according to the feasibility of approximation of the upper and lower cut edges of the airway. When more length was needed, a full drop was done (Fig. 1).

Postoperative feeding was secured through a nasogastric tube for one week. This period allows for care of possible postoperative dysphagia and achieves a full pharyngo-laryngeal rest to keep the anastomosis relaxed.

### Swallowing assessment

The assessment of swallowing was done by clinical evaluation of symptoms of swallowing dysfunction, Gugging swallowing screen (GUSS) and Fiberoptic Endoscopic Evaluation of Swallowing (FEES). Symptoms of swallowing dysfunction include choking, cyanosis or congestion during swallowing and wet voice. GUSS was used as a bed-side dysphagia screening that allows classification of patients’ swallowing abilities, and determines the severity of dysphagia [14, 15]. The severity of dysphagia was measured according to the total points score of the test where a score of 20 denotes normal swallowing abilities and no dysphagia, scores from 15 to 19 mean insignificant dysphagia with minimal risk of aspiration, scores 10–14 indicate moderate dysphagia with a sensible risk of aspiration, and scores 0–9 are rated as severe dysphagia with high risk of aspiration.

Instrumental swallowing evaluation using FEES was done and the risk of aspiration is determined according to the penetration aspiration scale (PAS) [16]. PAS score ranges from one to eight. Normal swallowing function equals PAS 1, laryngeal penetration corresponds to PAS 2–5 and aspiration corresponds to PAS 6–8. PAS 2 means that the contrast remains above the vocal cords and expelled while PAS 3 means that the contrast remains above the vocal cords also, but not expelled. PAS 4 means that the contrast touches the vocal cords and ejected while PAS 5 equals PAS 4 but the contrast is not ejected. PAS 6 means that the contrast reaches below the vocal cords but then expelled, PAS 7 means that the contrast reaches below the vocal cords but not ejected despite efforts while PAS 8 means that there is no even effort to expel the contrast.

The clinical and bed-side swallowing evaluations were done preoperatively and on postoperative follow ups one week and one month after the surgery, while the FEES was done preoperatively and one month postoperatively.

### Statistical analysis

Date entry and data analysis were done by SPSS version 26. Data were presented as number, percentage, mean, standard deviation. We used chi-square test to compare between qualitative variables, paired T-test in quantitative variables in the same group and independent sample t-test for quantitative variables between two groups. *P*-value considered statistically significant when *p* < 0.05.

## Results

A total of 71 patients suited the inclusion criteria and were included in the study. Group A included 46 patients who received mini laryngeal drop, and Group B comprised of 25 patients in whom a full laryngeal drop was performed. The mean age for group A was 24.89 ± 10.02 years while that for group B was 22.60 ± 13.21 years. Group A consisted of 32 (69.6%) males, and 14 (30.4%) females, whereas group B included 17 (68.6%) males and 8 (32.0%) females. Crico-tracheal resection with thyrotracheal anastomosis surgery was done in 24 patients (33.8%) (15 in group A and nine in group B) while lower trachea-tracheal resection operation was done in 47 patients (66.2%) (31 in group A and 16 in group B).

Clinical swallowing evaluation one week postoperatively showed that 37 patients (80.04%) from group A suffered from choking during swallowing in comparison to 100% of patients of group B with a statistical significance between both groups. While none of the patients of group A demonstrated wet voice, 21(84.0%) patients from group B exhibited wet voice which differed significantly between both groups. At the one-month postoperative follow-up, choking and wet voice were resolved in all patients of group A. However, choking was yet observed in 20 (80%) patients of group B and wet voice was also resolved in patients of group A while it remained in 5 (20%) patients of group B.

**Fig. 2:**
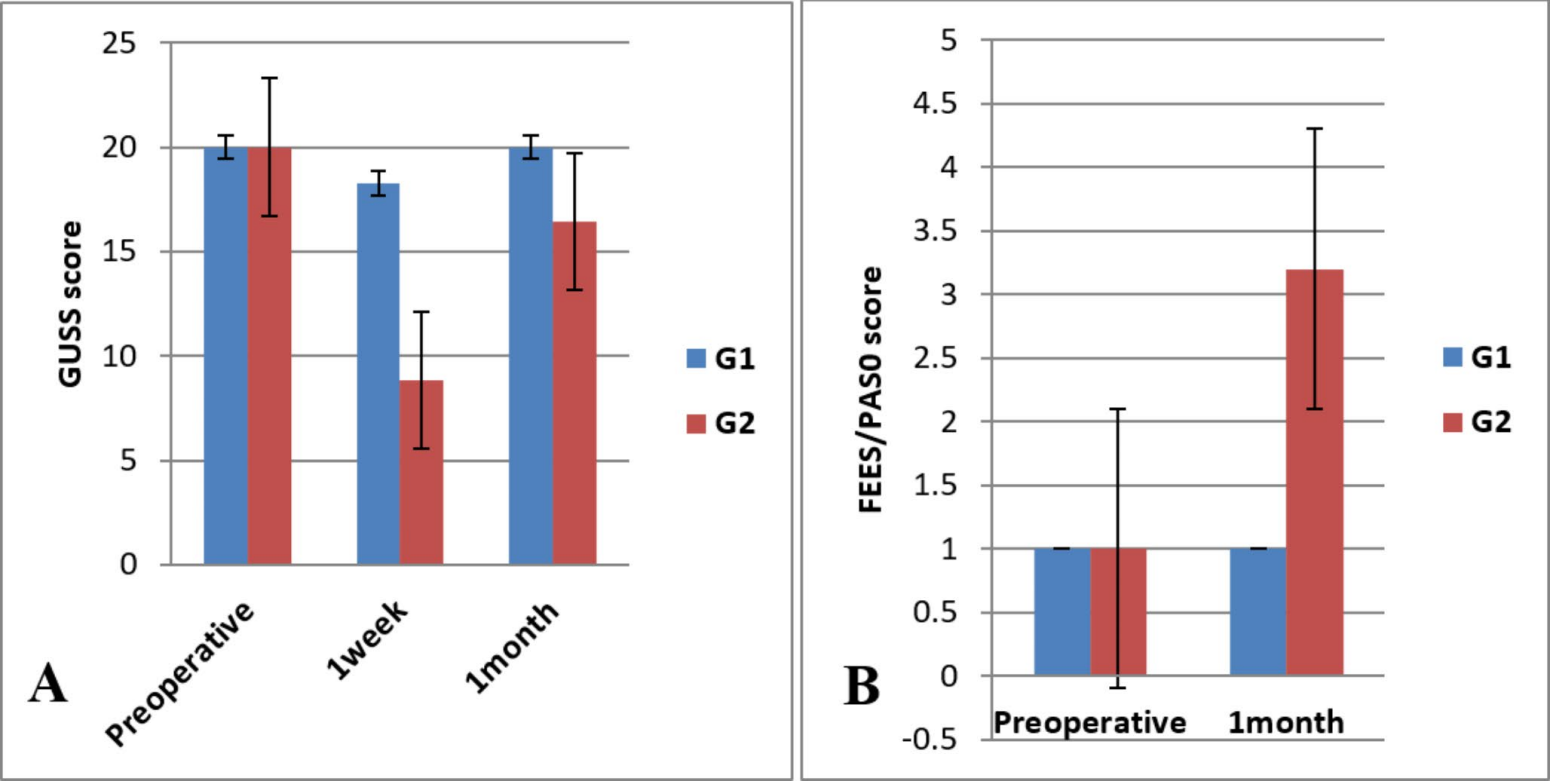
**A** Represents preoperative and postoperative Gugging swallowing screen (GUSS) test scores. G1 represents patients received mini laryngeal drop procedure (Group A) and G2 constitutes patients underwent full laryngeal drop (Group B). Significant differences between both groups were noted at one week and one month postoperatively. **B** Represents penetration aspiration scale scores in the study population. Notice the complete laryngeal drop procedure was associated with a more deterioration of the swallowing abilities at one month follow-up. Of note that higher GUSS scores correspond to near normal swallowing, but higher FESS scores mean more dysfunctional swallowing outcomes

Bed-side evaluation of swallowing function using the GUSS test preoperatively denoted normal swallowing abilities in all patients; 20 total score points. On one-week postoperatively, the mean scores differed significantly between both groups where it was 18 ± 1.32 in group A patients in comparison to 8.84 ± 5.18 in patients of group B (*p*-value < 0.001). This implies more deterioration of the swallowing function with full laryngeal drop. At the one-month postoperative visit, the mean scores of patients of group A increased to 20 points who regained normal swallowing abilities, meanwhile, it was significantly lower 16.44 ± 5.97 in patients of group B (*P* < 0.04*). Data of the preoperative and postoperative GUSS test scores are provided in Fig. 2.

Evaluation of patients using FEES showed that group A had full improvement of their swallowing abilities one month after the surgery with a mean penetration aspiration scale (PAS) score of 1 ± 0.0. On the other hand, patients in group B had significantly lower PAS scores; 3.20 ± 2.27. Figure 2 shows significant difference between preoperative and postoperative PAS scores in group B denoting deterioration of swallowing function postoperatively.

**Fig. 3:**
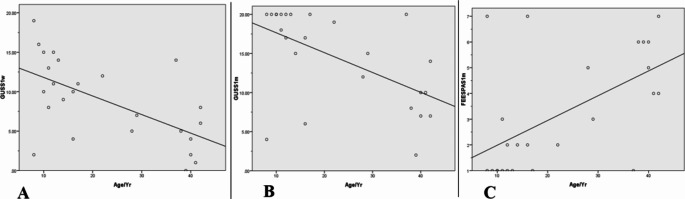
**A** A significant negative correlation between increasing patients’ age and the GUSS test scores one week postoperatively in G2 patients. **B** Shows also a significant negative correlation between increasing patients’ age and the GUSS test scores one month postoperatively in G2 patients. **C** Represents correlation between patients’ age and penetration aspiration scale scores one month postoperatively in G2 patients. Notice the worse penetration aspiration scale scores with increasing patients’ age

Unfavourable scores for both the GUSS test and PAS were associated with increasing patients’ age for patients underwent full laryngeal drop at all follow up time periods (*r* = − 0.600, *p* < 0.02 and *r* = − 0.56, *p* < 0.004), (*r* = 0.559 *p* < 0.004) for the GUSS test and PAS respectively. Interestingly, this was not noticed in patients underwent mini laryngeal drop. Correlations between patients age, the GUSS test scores and PAS scores for patients received full laryngeal drop are shown in Fig. 3.

The receiver operating characteristic curve (ROC) shows that GUSS test is highly sensitive in detecting swallowing dysfunction in patients undergoing PCTR. This was done by comparing the results of the GUSS test score to those of the FEES at one-month postoperative follow-up revealing a sensitivity of 95.8% and specificity 62.0% at cutoff point of 14.5.

## Discussion

Tracheal resection or crico-tracheal resection anastomosis has been established as the definitive surgical management for high grade post-intubation subglottic stenosis. The procedure had been continuously updated to achieve a relaxed tension free anastomotic closure especially in cases with challenging long stenotic segment. A major limitation in cases of tracheal resection is the length of the stenotic segment and subsequently, the tension created on the tracheal ends of the anastomosis. With resection lengths of less than 2–3 cm, no additional manoeuvres are usually needed to bring the two anastomotic ends together, but these situations are not often the case. In 1948, Rob and Belsey proposed a 2 cm length limit to be the extent for primary tracheal resection and reconstruction [17, 18].

The understanding of the surgical technique and the anatomical structures involved can result in a tension free and well-perfused anastomosis [19]. It is reported by Broussard and Mathisen that neck flexion and Para tracheal dissection can allow to resect up to 4 cm of the trachea without anastomotic complication [20]. Yet, patients with longer tracheal lesions handled by either limited surgical options or anastomotic complications following aggressive surgery. So, additional release techniques were employed to extend the tracheal resection limit up to 6 cm [1, 20, 21]. Rosen et al. [22] found that by using an appropriate laryngeal drop technique, about 6.68 cm, 13.3 tracheal rings or 65.5% of the total tracheal length can be removed without creating tension on the anastomosis.

A different tracheal release procedures were reported as suprahyoid, infrahyoid and paracardial release manoeuvres [20]. These release manoeuvres can affect longitudinal pharyngeal muscles that assist in pharyngeal expansion and constriction during swallowing. Other possibly affected muscles are the suprahyoid muscles including the digastric, geniohyoid, stylohyoid, and mylohyoid, as well as the infrahyoid muscles such as the omohyoid, sternohyoid and thyrohyoid, which are responsible for elevation and depression of the hyoid bone, respectively, during swallowing.

Affection of these muscles had been associated with dysphagia in the early postoperative period following tracheal resection [23, 24]. While recent studies have identified various surgical factors that contribute to dysphagia following airway resection surgery, few reports concerned with discussing the difference in swallowing outcomes according to the method of laryngeal release used [25].

Dedo and Fishman [26] described the infrahyoid laryngeal release in 1969. The technique involved dividing the thyrohyoid muscles, thyrohyoid membrane and the superior horns of the thyroid cartilage. Grillo et al. reported that 2.5 cm of proximal tracheal mobilization can be achieved with infrahyoid laryngeal release in a series of 49 cases. This was associated with swallowing problems in about 1.2% of cases [1]. Maassen [27] also reported some transient swallowing problems after infrahyoid laryngeal release.

Suprahyoid release was discussed in 1974 by Montgomery et al., [28] in which the mylohyoid and geniohyoid muscles were detached from body of the hyoid bone, which was then separated from the greater and lesser cornu. This procedure allows for pulling down the proximal trachea by about 2–3 cm [29]. Transient swallowing dysfunction occurred in 17 (32.2%) patients reported by Mohsen et al., exclusively in patients who underwent laryngeal release. Notably, this dysfunction was temporary and relieved in the early postoperative period [30].

In the current study, a comparison between two techniques of infrahyoid laryngeal drop was conducted. These two techniques are considered mini or full drop after the preservation or fracture of the superior thyroid horn bilaterally. The final swallowing outcomes were significantly better in the mini release group with greater severity of swallowing difficulties in the full group. In a previous study, the length of the resected trachea and hence the need to do more laryngeal release did not cause dysphagia or affect diet after the surgery [11].

In the current study, a correlation between older patients’ age and worse swallowing outcomes was found in the group having a full laryngeal drop. Christopher et al., concluded similar results in their study in which there was an association between patient age and swallowing scores, suggesting that the age could increase the severity of dysphagia after tracheal resection and delay the return to normal preoperative diets [11]. This can be explained by their lower baseline functional status with expected greater postoperative functional deficit after disruption of the normal swallowing mechanism during surgery. Given the existing literature, it has been found that disruption of soft tissues during laryngeal drop in tracheal resection in patients of increased age contributes to greater postoperative affection of swallowing and prolonged dysphagia. So, patients of advanced age should be counselled to expect swallowing difficulties especially if a full laryngeal drop was implemented [31–33].

The current study has some limitations. Its retrospective nature made data collection limited to the previously available recorded variables. Also, swallowing outcomes were evaluated in the immediate post-operative period (one month). The fact that makes the long-term functional outcomes still unclear. Therefore, future studies should be performed with structured observation and a long-term follow-up period. Additionally, our results revealed that GUSS test has a high sensitivity in evaluation of swallowing outcomes. This is of a notable importance as GUSS test is easier to implement than FEES. However, this result, being based on a retrospective study, needs to be furtherly investigated using prospective comparative studies to have a more accurate validation.

## Conclusion

In this retrospective cohort study, cases with mini infrahyoid laryngeal release had significantly better swallowing outcomes and full resolution of dysphagia in comparison to full laryngeal release. Also, full laryngeal release is associated with delayed resolution of swallowing difficulty in older patients. This point should be considered during preoperative patient selection and counselling.
